# Human capital and socialism builders: a happy marriage? Analysing the construction of ‘high-level talent’ in Chinese higher education policy

**DOI:** 10.1007/s10734-024-01379-8

**Published:** 2024-12-21

**Authors:** Zhiyun Bian

**Affiliations:** School of Education, Durham University, Durham, UK

**Keywords:** Critical discourse analysis, Fairclough, Higher education, Educational policy, China, Double First-Class

## Abstract

This paper employs critical discourse analysis to investigate the construction of ‘high-level talents’ within China’s Double First-Class Project, an educational initiative implemented in 2015 to establish first-class universities and disciplines and cultivate high-level talents. The study examines the juxtaposition of human capital discourse and the political concern of ‘socialism builders and inheritors’ as articulated in key policy texts, including President Xi Jinping’s speeches and various government documents. It investigates how the global discourse of human capital has been recontextualised within Chinese higher education policy, highlighting the tensions and negotiations between economic objectives and ideological imperatives. The findings reveal a hybrid discourse—‘high-level talents with Chinese characteristics’—that reflects the Chinese Communist Party’s strategy in crafting its narrative to negotiate compliance with global policy discourses while strengthening its governing power in an increasingly globalised, economic, and individualising world. This study contributes to the understanding of how global educational ideologies are localised, offering insights into the implications for students’ educational choices and identities within the framework of China’s socio-political landscape.

## Introduction

The relationship between education and development has been a central concern in global educational policies. National and international power players constantly strive to influence and control how development is conceptualised and achieved (Phillips & Schweisfurth, 2014). The idea of human capital, which regard education as an investment for individuals and nations, is influential in understanding the relationship between education and economic development. As a globally dominant idea, it has gained renewed prominence in educational policy discourses associated with neoliberalism, characterised by deregulation and market-oriented reform (Marginson, 2019). Higher education (HE) has been increasingly recognised for its significant role in generating human capital to create a new knowledge economy and achieve success in global competition.

In East Asia, (higher) education has long been seen as a tool for political socialisation, that is, to shape and sustain political systems (Lall & Vickers, 2009). During President Xi Jinping’s (hereafter President Xi) era in China, education’s ideological and political dimensions have been further strengthened, alongside a prominent emphasis on HE serving economic ends (Han & Xu, 2019; Vickers & Zeng, 2017). The heavy ideological and political imprints in HE have raised questions about the role of HE institutions in negotiating the dual goals of accumulating human capital and fostering ‘socialism builders and inheritors’ (*shehuizhuyi jiebanren* 社会主义接班人)—socialist citizens with Chinese characteristics who are expected to uphold socialist values and ensure the continuity of socialist governance and ideology (Xi, 2014). This term is commonly used in Chinese political discourse.

In China, the Double First-Class Project was introduced in 2015 to build world-class universities and first-class disciplines, as well as to cultivate ‘high-level talents’ (*gaoshuiping rencai* 高水平人才) with Chinese characteristics (The State Council, 2015). A total of 147 universities have been included in this initiative, representing about 5% of Chinese universities (Liu, 2018). One of the overall aims of this initiative is to cultivate ‘high-level talents’:

Develop a batch of high-level world-class universities and disciplines… and make them a significant source of knowledge discovery, scientific and technological innovation, advanced thoughts, excellent culture, an important cradle of high-level talents who can play a significant role in supporting the national innovation-driven development strategy, serving socio-economic development, inheriting, and promoting Chinese excellent traditional culture, and following the Core Socialist Values (The State Council, 2015).

This paper aims to critically analyse the construction of ‘high-level talents’ within the policy discourses of China’s Double First-Class initiative. Specifically, it explores the representation of HE students as human capital, juxtaposed with China’s socialist ideology. It seeks to address the following questions: (1) How has the global discourse of human capital been ‘recontextualised’ in Chinese HE policy discourses to represent ‘high-level talents’? (2) How are potential conflicts between the human capital and ‘socialism builders’ narratives negotiated within these policy discourses?

To answer these research questions, Norman Fairclough’s (2003) critical discourse analysis (CDA) is adopted to analyse the selected policy documents—six President Xi’s speeches and four government documents. The findings offer important insights into the Chinese Communist Party’s (CCP) strategy in crafting its narrative to negotiate with the pressure of complying with global educational policy discourses and to strengthen its governing power in an increasingly ‘globalised, economistic and individualising’ world (Arnott & Ozga, 2010, p. 335).

This article is structured as follows: It begins by placing the Double First-Class University agenda in the socio-political context in China. It then introduces the idea of human capital as a globally dominant discourse that has travelled and embedded in Chinese HE policy discourses. The remaining part of the paper outlines the methodology and the main analytical categories used. Finally, this paper analyses the findings of CDA and discusses its contributions and implications for further studies.

## Human capital as a social imaginary

This article views the articulation of ‘human capital’ as a globally dominant discourse, or social imaginary, that has travelled and become embedded in Chinese HE policy discourses (Ozga & Jones, 2006). Building on the work of Jones and Alexiadou (2001) and Ozga and Jones (2006), ‘travelling policy’ refers to the activities of supranational and transnational agency activity, while ‘embedded policy’ describes how these global agendas interact with and adapt to local (national, regional or local) contexts and priorities. This perspective acknowledges that national and local contexts remain influential in mediating or translating global policy agendas (Ozga & Jones, 2006).

The idea of human capital originates from human capital theory (HCT), which gained prominence in the 1960s through the work of neo-classical (‘Chicago School’) economists such as Gary Becker (2009) and Theodore Schultz (1961, 1971). HCT posits that education is not merely consumption but an investment that yields returns for both individuals and nations. To simplify and summarise the line of its assumption, for individuals and their families, investing in education enhances productivity and leads to higher earnings. At the national level, improving the quality of the labour force through education promotes economic growth (Marginson, 1989; Tan, 2014).

In recent years, HCT has been closely linked with the rise of the knowledge economy (Marginson, 1989; McCowan & Unterhalter, 2015). Knowledge and education are increasingly viewed as business products, with innovative intellectual outputs and services expected to yield high-value returns (Ball, 2017). Influential international agencies such as the World Bank and OECD have played a key role in promoting human capital, advocating lifelong learning and the development of knowledge economies (Berkovich & Benoliel, 2020). HE, as a key component of the national innovation system, is emphasised for its research output and its role in producing highly skilled knowledge workers.

The idea of human capital has also been influenced by the ‘triumph of the neo-liberalism imaginary’ in ‘global policyspeak’—the ‘international flows of policy discourses’ (Ball, 2012, p. 2; Ball, 2017, p. 2; Novoa, 2002). In HE, this trend is evident in policies favouring privatisation, marketisation, and cost-sharing with public systems and individuals, with a renewed emphasis on the private rates of return of HE (Égert et al., 2019; Marginson, 1989). Lingard (2009, p. 18) provided a compelling account of the contemporary trend:

Globalisation as experienced over the past thirty years or so has been neoliberal globalisation, an ideology which promotes markets over the state and regulation and individual advancements/self-interest over the collective good and common well-being. We have seen a new individualism, with individuals now being deemed responsible for their own ‘self-capitalism’ over their lifetimes. Common good and social protection concerns have been given less focus and the market values over the state, with enhanced market or private sector involvement in the workings of the state.

This quote is particularly useful when contrasted with the Chinese HE sector, where the state has never ‘stepped back’ (Han & Xu, 2019).

Following these trends, the meaning of human capital has further evolved and expanded. Initially understood as knowledge, skills, and competence directly linked to economic productivity, it has been broadened to encompass the idea that ‘my human capital is me, as a set of skills and capacities that is modified by all that affects me and all that I affect’ (Feher, 2009, p. 26). Previously conceived as workers exchanging their labour power as ‘a productivity commodity’ for material gain, individuals are now seen as ‘managers of a portfolio of conducts’ across all aspects of life or as ‘entrepreneurs’ with a particular set of saleable skills and attributes (Feher, 2009, p. 30; Sellar & Zipin, 2019, p. 574). In other words, they have become investors in themselves.

As the meaning of human capital has expanded, there has been a tendency to incorporate non-cognitive attributes, traditionally associated with moral domains, into ‘human capital portfolios’ (Sellar, 2015). For example, Bowles et al. (2001) found that certain non-cognitive skills, referred to as ‘incentive-enhancing preferences’ are often rewarded by labour markets. Similarly, Sellar and Lingard (2014) observed that the OECD introduced the concept of ‘wider human capital’, which includes attributes that enable individuals to build, manage, and deploy basic human capital, such as productive capacities and skills (p. 576). This broader definition emphasises the importance of ‘motivational characteristics’, including future orientation.

While the discourse of human capital has been influential in understanding the relationship between education and economic growth, it has been criticised for being shaped by the historical development of Western, industrialised, capitalist nations and for overlooking the distinct contexts of developing countries (Harber, 2014, p. 111). As McCowan and Unterhalter (2015) noted, it often assumes capitalism as the ideal system, frames economic growth as the primary indicator of development, and prescribes a universal development path for all nations. The next section examines the role of HE as ‘a political tool’ in China—rather than merely an economic one—to advance national development under a different ideological framework: socialist forms of development (Lall & Vickers, 2009).

## Education as a political tool in China

Education has long been regarded as a key instrument for political socialisation, defined as ‘the learning of preferences and predispositions towards political values and attitudes’, such as citizenship education (Harber & Mncube, 2012, p. 28). A distinguishing feature of socialist education systems, compared to capitalist models, is a more explicit form of ‘political indoctrination/socialisation’, described as ‘an attempt to intentionally inculcate values and beliefs as facts or truths’ (Harber & Mncube, 2012, p. 28). It primarily aims to shape the next generation of socialist citizens (Harber, 2014, p. 118).

For the CCP, ideological and political education has long been a central concern, encompassing political indoctrination, patriotic education, and moral instruction (Jiang & Xu, 2013, p. 72). This form of education is considered crucial for cultivating 'socialism builders and inheritors’ (*shehuizhuyi jianshezhe he jiebanren*社会主义建设者和接班人) and fostering all-round development in ‘morality, intellectuality, physics, and aesthetics’ (Xi, 2014; Zhao, 2013). Its primary aim is to instil patriotism, discipline, and loyalty to the Party (Vickers & Zeng, 2017). A key theme is the ‘politicalised’ form of moral education that emphasises socialist ideals, ethics, and patriotism (Jiang & Xu, 2013). For example, the core socialist values, introduced by President Xi in 2012, consist of 12 values across three dimensions: national, social, and individual. At the individual level, these values include ‘patriotism’, ‘dedication’, ‘integrity’, and ‘friendship’ (Xi, 2014, p. 205). Vickers (2010) argued that the heightened focus on moral education following China’s shift to a market economy serves to counteract consumerism and individualism, reinforcing social cohesion in a rapidly changing world.

Ideological and political education in HE institutions has been further strengthened during President Xi’s era (Vickers & Zeng, 2017). Although the state has gradually reduced direct intervention in HE institutions since the 1980s, ideological and political education remains firmly under state control. For instance, all state-run HE institutions are required to offer compulsory ideological courses (Jiang & Xu, 2013). Beyond compulsory courses, ideological education includes activities such as Communist Youth League gatherings and patriotic ceremonies (Vickers & Zeng, 2017). Furthermore, Chinese academics, especially those in the Humanities and Social sciences, are closely monitored by the central government and are expected to contribute to ‘propagating and sustaining the ideological discourses that impart legitimacy to the Chinese government’ while pursuing international excellence with a ‘heightened awareness of ideological correctness’ (Gao & Zheng, 2020; Liu et al., 2019, p. 558).

One of the reform measures of the Double First-Class initiative was the introduction of a performance-based assessment system, replacing the earlier ex-ante assessment system, which relied on ‘quantitative and objective criteria such as staffing, facilities and research’ (Han & Xu, 2019, p. 941; Mok, 2002). The new system employs continuous performance evaluations and dynamic adjustments to regulate funding allocation (Han & Xu, 2019). Notably, strengthening and improving ideological education in HE institutions have become a significant accountability measure within this performance-based assessment system (MoE, Mof, and NDRC, 2020). These measures have raised growing concern about their impact on HE autonomy (Han & Xu, 2019; Liu, 2018). The examination of the marriage between human capital and socialism builders takes place against the backdrop of these reforms and the seemingly contradictory expectation that HE serves as both economic and political tools.

## The methodological approach

This study adopts Norman Fairclough’s (2003, 2010) critical discourse analysis (CDA) to analyse nine policy documents—six President Xi’s speeches and four government documents (see Table 1). The aim is to investigate how the CCP government has ideologically invested in the discourses of high-level talents to enhance its governing power in the age of neoliberal globalisation. CDA is particularly useful for identifying how power relations and inequalities produce and perpetuate ‘social wrongs’, with a focus on discursive aspects (Fairclough, 2003, p. 8).

**Table 1:** Overview of the selected texts

	Full title
President Speech 1	Xi, J., *Towards World-Class Universities and Disciplines*. Speech given at a national conference on education in political philosophy at institutions of higher education, 7 December 2016. (Xi, 2017, pp. 406–410)
President Speech 2	Xi, J., *Achieving Rejuvenation is the Dream of the Chinese People*. Speech made when visiting the exhibition ‘The Road to Rejuvenation’. 29 November 2012. (Xi, 2014, pp. 30–33)
President Speech 3	Xi, J., *Realise Youthful Dreams.* Speech to outstanding young representatives from all walks of life. 4 May 2013. (Xi, 2014, pp. 44–50)
President Speech 4	Xi, J., *Build China into a World Leader in Science and Technology*. Speech at the joint session of the National Conference on Scientific and Technological Innovation. 30 May 2016. (Xi, 2017, pp. 292–303)
President Speech 5	Xi, J., *Make China a Global Centre for Science and Innovation*. Speech at the joint session of the 19th Meeting of the Members of the Chinese Academy of Sciences and the 14th Meeting of the Members of the Chinese Academy of Engineering. 28 May 2018. (Xi, 2020, pp. 287–298)
President Speech 6	Xi, J., *Carry on the Legacy of the May 4th Movement, and Be Worthy of the New era*. Speech at a conference marking the centuary of the May 4th Movement. 30 April 2019. (Xi, 2020, pp. 387–392)
Government Document 1	The State Council., 2015. *Overall Plan for Co-ordinately Advancing the Construction of World-Class Universities and First-Class Disciplines*
Government Document 2	Ministry of Education, Ministry of Finance and National Development and Reform Commission. 2018. *Guiding Opinions on Speeding Up the Construction of ‘Double First-Class’ Universities*
Government Document 3	Ministry of Education, Ministry of Finance and National Development and Reform Commission. 2020.* Opinions on Strengthening and Improving Ideological and Political Work in Higher Education Institutions under New Circumstances*
Government document 4	Ministry of Education, the Academic Degrees Committee of the State Council. 2017. *The 13th Five Year Plan for the Development of Degree and Postgraduate Education*

The term ‘discourse’, in its concrete aspect, refers to particular ways of representing aspects of the world (Fairclough, 2003). In Fairclough’s (2003) view, discourses not only reflect the perceived reality but also serve as a means of imagining alternative worlds, often linked to efforts to change the current state of the world (p. 124). For example, the discourse of human capital frames HE as an ‘investment’ for both individuals and nations. Discourse interacts with other elements of social practices, meaning it can be operationalised into practice, enacted as social action, internalised as new identities, and physically materialised (ibid.). For example, the representations of the ideal HE student, such as the notion of ‘self as human capital’, might shape students’ HE choices, and influence how they see themselves and engage with others (Gillies, 2011, p. 228).

CDA’s transdisciplinary theoretical framework fosters dialogue between sociolinguistics and social theories, a process Chouliaraki and Fairclough (1999) termed ‘theoretical translation’. A key concept in this study is *recontextualisation* (including re-structuring & re-scaling), originally developed by Bernstein (2003). It refers to how discourses move spatially and temporally between different contexts, undergoing transformations shaped by the relationships and differences between them (Wodak & Fairclough, 2010, p. 22). This can involve the ‘colonisation’ of one field or institution by an external discourse or an ‘appropriation’ of an external discourse, where particular social agents within the recontextualising field might strive to incorporate it into their own strategies (Fairclough & Fairclough, 2012, p. 83).

The concepts ‘equivalence and difference’ are borrowed from post-Marxist theorists Laclau and Mouffe (1985), who adapted Antonio Gramsci’s theory of hegemony into discourse analysis. These logics are involved in social processes of classification, where texts constantly combine some elements (in their textual forms as words and expressions) and divide others to create semantic relations of equivalence and difference (Chouliaraki & Fairclough, 1999). This meaning-making process plays a crucial role in the political struggle for hegemony (Fairclough, 2003, p. 101).

When weaving together different discourses, texts may establish ‘dialogical or polemical relations’ between discourses or mix them into a hybrid discourse (Fairclough, 2003, p. 128). Even in seemingly incompatible discourses, the strategy of ‘legitimation’ can be employed to create a unified narrative, though a hierarchical order may persist within the integrated discourses (ibid.). For example, the New Labour government sought to legitimate the social cohesion discourse through a neoliberal lens, with the latter taking the lead (Fairclough, 2000).

### Text selection and the analytical categories

This study adopts a purposive sampling strategy. As Machin and Mayr (2012) suggested, texts are often purposely selected based on ‘the interest of the analyst, where perhaps they have observed ideology in operation’ (p. 206). Given that CDA prioritises in-depth explanation over generalisation, it is not uncommon for analysts to select ‘only a small number of texts’ (Cassell & Symon, 2004; Machin & Mayr, 2012, p. 8; Sengul, 2019).

For this study, relevant government documents were sourced from the State Council online policy database (http://www.gov.cn/zhengce/zhengcewenjianku/index.htm) and the Ministry of Education’s website (http://www.moe.gov.cn), using ‘Double First-Class’ (*shuangyiliu* 双一流) as a keyword. Additionally, speech transcripts were obtained from the three-volume collection—*Xi Jinping: The Governance of China* (Xi, 2014, 2017, 2020), published by Foreign Language Press.^1^ These government documents and presidential speeches were identified and scrutinised for their relevance in outlining key policy priorities for cultivating ‘high-level talents with Chinese characteristics’. The texts were scanned, and key extracts were selected for in-depth analysis focused on the definition of ‘high-level talents’, barriers in constructing ‘world-class universities and disciplines’, proposed measures for establishing these institutions and cultivating high-level talents, and the underlying assumptions about ‘useful’ disciplines.

The analytical categories used for policy document analysis are outlined below. They were selected based on the analytical tools provided by Fairclough (2003, pp. 191–194) and the critical HE policy discourse analysis framework developed by Hyatt (2013).

1. ‘Macro’ semantic and societal levels of the policy texts:
  - Interdiscursive analysis: analysis of which genres, discourses, and styles are drawn upon, and how they are articulated together (Fairclough, 2003, p. 238)
  - Semantic relations: relations of equivalence and difference
  - Legitimation
  - Evaluation (including assumptions, modality)
2. ‘micro’ lexico-grammatical levels (e.g. pronouns, voice)

## The discourse of science and technology human capital

The neoliberal discourse of human capital, as discussed earlier, is a globally dominant or ‘travelling’ educational policy discourse. At the national level, HE is viewed as an ‘investment’ crucial to national economic development. The CDA results indicate that this human capital discourse can be prominently distinguished in Chinese policy texts. However, it has been reshaped into a local discourse of science and technology human capital, positioning HE as an engine for producing high-level talents in these fields. This discourse is a key resource leveraged by the CCP government to pursue the Chinese Dream of building ‘China into a world leader in science and technology’ (Xi, 2017, p. 292). Key extracts illustrating this discourse are provided below (emphasis added):

1. Economic globalisation is superficially exemplified by the flow of products, capital and information across the world. However, behind such a flow is the propelling force of human talent and scientific and technological innovation capability (President Speech 4).
2. To become scientists is the dream of countless Chinese children. We should make scientific work so attractive that they would aspire to pursue it. Let us give the children’s dreams the wings of science and technology. Let China’s future field of science and technology be filled with talent. Let them be the stars shine in the future science sky! (President Speech 5).

### Semantic relations of equivalence and vocabulary

Vocabulary is a powerful tool for distinguishing discourses, as it ‘words’ or ‘lexicalises’ aspects of the world from particular perspectives (Fairclough, 2003, p. 129). In extract 1, economic globalisation is presented as a fact, with the ‘propelling force’ behind the ‘flow of products, capital, and information across the world’ identified as ‘human talent’ and ‘scientific and technological innovation capability’. The former is implicitly reworded as the latter, establishing a new relation of equivalence between concrete entities (talents or human resources) and abstract concepts (innovation, particularly science, and technological innovation). This rewording, or the setting up of a new relation of equivalence, creates a local texturing of semantic relations, a part of the work of social agents (mainly the CCP government) in making meaning.

Extract 2 provides another example. Except for President Xi’s (or the CCP’s) voice, it brings out Chinese children’s voices, but it does so in an abstract, universal way. Becoming a scientist might be the dream of some children, while others may aspire to different careers. However, the ‘particular’ (particular interests, thoughts, representations) is represented as ‘universal’ (‘countless’). Fairclough (2003) describes this universalisation of a particular representation—the action to negotiate, reduce, or suppress difference—as a hegemonic strategy to suppress differences and project certain particulars as universal (p. 41).

The value assumption in extract 2 is clear—children should ‘aspire to’ careers in science and technology. The pronoun ‘we’ assumably refers to the CCP government, but it may also encompass parents and teachers, implying a collective responsibility to ‘make scientific work attractive’. When such ideas become shared assumptions, they gain considerable social importance. As Fairclough (2003) argues, exercising social power and hegemony involves shaping the ‘common ground’ of accepted beliefs. Implicitness and assumptions, therefore, often reflect the ideological work of texts, as seen here in the effort to position science and technology professions as more desirable than others, particularly those offering private rather than social returns.

### Evaluations and value assumptions

A key linguistic feature highlighting the role of the human capital discourse in the policy texts is the use of (value) assumptions, which are both discourse-relative and discourse-specific. Linked to assumption is evaluation, which distinguishes what is deemed desirable or undesirable by the author(s). For example,

3. The forewarning system should be established to caution against disciplines that have long been of low quality, do not align with national socio-economic development demands, or have produced an oversupply of graduates (Government Document 4).

In extract 3, the noun phrase ‘low quality’ and the process noun ‘overproduction’ are clear negative evaluation markers, while ‘in line with national socio-economic development demands’ is assumed to be implicitly desirable without evaluation markers. This extract is discourse-specific, tied to human capital discourse, with its emphasis on labour market needs and national economic development.

This extract is also highly abstract, with the social actor responsible for the ‘overproduction’ left ambiguous. Disciplines are framed as lagging behind socio-economic demands, making them the primary targets of reform, rather than social actors like the government. National socio-economic development demands are presented as an objective reality, beyond the influence of any ind social actors, including the CCP government. This framing of the causal relation constrains the possible policy options and makes the proposed reform of the disciplinary system appear inevitable.

Other extracts, summarised in Table 2, are drawn from Government Document 2 and 4. They reflect the CCP’s evaluation regarding disciplinary system reform, talent training, and preferred student abilities. These evaluations and value assumptions indicate that the human capital discourse is a central framework in the Chinese HE policy texts for constructing the image of high-level talents. However, one entry (marked with *) is an exception, linking the construction of high-level talents to another master discourse—the discourse of socialism builders and inheritors, which is discussed in the next section.

**Table 2:** Evaluations of the disciplinary system: desirable and undesirable

Desirable	
The disciplines and subjects	Urgently demanded by national socio-economic development in the foreseeable future
	Serving national strategies and meeting the urgent needs of society and the people
	Emerging and inter-disciplinary subjects of great practical significance
	The development of philosophy and social science with Chinese features such as Marxist subjects and those inheriting outstanding Chinese traditional cultures*
Talent training system	Labour-market-oriented
	Professional postgraduate degree programs with a close link to employability
	Combining the labour market demands and higher education knowledge acquirement
Student	Innovative ability and (the labour market) applied ability
	A balance between book knowledge learning and applied capability
	Career development ability, career adaptability, and entrepreneurship after graduation
Undesirable	
The disciplines and subjects	Not in line with national socio-economic demands
	Having nothing so much to do with social development
	Generating an overproduction of graduates
	Have long been of low quality
Student	Too much emphasis on academic performance and research outputs

## The nationalist discourse of socialism builders and inheritors

The discourse of socialism builders and inheritors focuses on equipping HE students—the new generation of Chinese citizens and potential high-level talents—with ‘correct’ and ‘strong moral characters’ to carry on the cause of Chinese socialism under the leadership of the CCP (Xi, 2017, p. 374). Key extracts that draw upon this discourse include (emphasis added):

4. China’s universities and colleges shoulder the responsibility of training new generation of socialism builders and inheritors with all-round development of morality, intelligence, physics and aesthetics. Therefore, these higher educational institutions must not waver from the current political direction. Building a strong moral character (lideshuren 立德树人: to promote a code of ethical conduct and to cultivate talent) is the fundamental task of universities and colleges. Only those who can provide society with first-class talents can become world-class universities (President Speech 1).
5. Chinese universities and colleges are institutions founded on Chinese socialism under the leadership of the CCP. To build better universities and colleges, we should follow the guiding role of Marxism and thoroughly carry out the CCP’s educational policies. We should promote and practice the Core Socialist Values, and guide teachers and students to be firm believers, active preachers, and model practitioners of these values (President Speech 1).
6. Chinese universities and colleges are institutions founded on Chinese socialism under the leadership of the CCP. To build better universities and colleges, we should follow the guiding role of Marxism and thoroughly carry out the CCP’s educational policies. We should promote and practice the Core Socialist Values, and guide teachers and students to be firm believers, active preachers, and model practitioners of these values (President Speech 1).

### Semantic relations of equivalence

The analysis of semantic relationships between words reveals how the discourse of socialism builders and inheritors represents ‘world-class universities’ and ‘first-class talents’. A chain of equivalences is set up between the concept of ‘world-class universities’ and (a) ‘outstanding brain banks’, (b) ‘institutions founded on Chinese socialism under the leadership of the CCP’, and (c) ‘better universities and colleges’ following ‘the guiding role of Marxism’ and ‘carry(ing) out the CCP’s educational policies’. Semantically, these descriptions (a–c) are (*co*-)*hyponyms* of ‘world-class universities’, reflecting the CCP government’s expectations of ‘world-class universities’.

Similarly, a chain of equivalences exists between ‘first-class talents’ (also referred to as ‘high-level talents’) and (a) ‘socialism builders and inheritors with all-round development’ not only in ‘intelligence’ but also in ‘morality’; (b) ‘wholesome and capable individuals’ with enhanced ‘moral standards and political awareness’ as well as refined ‘character and manners’; (c) ‘firm believers, active preachers, and moral practitioners’ of *core socialist values*; and (d) ‘talents of the new era who can take on the responsibility of national rejuvenation’. The focus on morality and political awareness explicitly invokes the discourse of socialism builders and inheritors.

## Interdiscursive analysis: a hybridisation of the two master discourses

Discourses are often interwoven within texts rather than opposed. As Fairclough (2003) explained, interdiscursivity refers to the particular mix of genres, discourses, and styles within texts. This paper focuses on interdiscursive analysis, particularly in its discourse aspect—discourse mixture or hybridity. This involves identifying which discourses are drawn upon and how they are articulated together. The discourse of high-level talents can be understood as a new discourse that emerges from a combination of existing discourses in a particular way or as ‘a novel articulation of the old’ (Fairclough, 2003, p. 127). The following excerpts illustrate how the discourse of high-level talents is constructed through the hybridisation of the two master discourses:

7. Ideological and political education is essentially designed to help people adopt the right kind of mindset… to facilitate their all-round development towards high-level talents with both competence and high-calibre (President Speech 1).
8. Young people must have professional competence. The competence and capacities of young people will have a direct influence on the realisation of **the Chinese Dream** (President Speech 3).
9. Young people must orient yourselves to modernization, the global world and the future, have a sense of urgency in updating knowledge… assiduously study theories while enthusiastically developing skills, and constantly enhance your competence and capabilities to meet the development needs of the new era and the requirements of the labour market (President Speech 3).

The construction of high-level talents with both ‘competence’ and ‘high-calibre’ (*decaijianbei*德才兼备) can be seen to do the ‘relational work’ of blending the two master discourses. As Fairclough (2003) notes, vocabulary is a distinguishing feature of discourse, and even a single word can reveal the specific discourse at play in a text. Within the discourse of human capital, the primary concern is young people’s ‘competence’ (*cai*才). The desired ‘competence and capabilities’ are those aligned with ‘the requirements of the labour market’, and considered instrumental to ‘the realisation of the Chinese Dream’.

10. The League should lay a solid intellectual basis for all young Chinese with **the Chinese Dream**, educate and help them to establish a correct world view, outlook on life and sense of values, always love our country, our people and our nation, and firmly follow the Communist Party along the Chinese path (President Speech 3).

In contrast, within the discourse of socialism builders and inheritors, ‘high-calibre’ (*de*德) is a central term. Unlike ‘competence’, which highlights technical and professional abilities, ‘high-calibre’ refers to the moral character of young people, focusing primarily on their ethical and ideological development. The excerpts above underscore the significance of the discourse of socialism builders and inheritors. Notably, the ‘Chinese Dream’ appears in both master discourses, suggesting it serves as a bridge term linking the two master discourses (see Table 3).

**Table 3:** The hybridisation of two master discourses: ‘high-level talents with both competence and high-calibre’

Keywords/phrases (human capital)	Bridging term	Keywords/phrases (socialism builders and inheritors)
Competence, skills, capabilities, abilities	The Chinese Dream: ‘Small I’ (individual) ↓ ‘Big we’ (collective)	Love our country, our people, our nation
Individual ideal, ambition, and dream (self-admiration)		Follow the CCP
The labour market		Correct worldview, life outlook, sense of values

11. Every individual has an ideal, ambition and dream… In my opinion, achieving the rejuvenation of the Chinese nation has been the greatest dream of the Chinese people … History shows that the future and destiny of each and every one of us are closely linked to those of our country and nation.^2^ One can do well only when one’s country and nation do well (President Speech 2).
12. Young people have different life goals and different career choices. However, we should cultivate their awareness of China’s historical mission and inspire them, through the Chinese Dream, to actively integrate the pursuit of their own dreams into the causes of our country and our people, and to forge ahead as pioneers. Any self-admiration which is far away from the needs of one’s country or the interests of the people will fall into a narrow world (President Speech 6).

As discussed earlier, the neoliberal discourse of human capital frames individuals as *homo economicus*—rational, utility-maximising individuals with individualism as an underlying value (Tan, 2014). In contrast, collectivism, nationalism, and social cohesion are central values for socialism builders and inheritors. Within the hybrid discourse of high-level talents, the individual’s dream and destiny are inextricably linked to those of the country and nation. This suggests that, in the process of hybridisation, the human capital discourse is reshaped through the lens of socialism builders and inheritors. Collective success (‘one’s country and nation do well’) is positioned as the prerequisite for individual success (‘one can do well’). This can be seen as the CCP’s strategy to persuade young people to view their self-interest from a nationalist perspective (Vickers & Zeng, 2017). In other words, in the pursuit of individual success, one should first meet the fundamental moral requirement—patriotism. When patriotism is framed as a non-cognitive trait that contributes to individual success, it becomes legitimate to incorporate it into the expanded meaning of human capital, alongside other moral traits such as ‘dedication’ and ‘integrity’, as outlined in the core socialist values.

### The hierarchy of discourses

Integrating patriotism into the expanded definition of human capital indicates a hierarchical relationship between the two master discourses. The preeminence of the discourse of socialism builders and inheritors is evident through an analysis of evaluation and modality in the following excerpts:

13. Chinese features and world-class status are the core, to build strong moral character is the fundamental task, to support innovation-driven (national development) strategy and to serve the economic development are the orientation (President Speech 1).
14. Building strong moral character should be taken as the central task of higher education (President Speech 1).

According to Fairclough (2003), evaluation and modality markers indicate an author’s commitments regarding ‘what is true’ and necessary, ‘desirable or undesirable’, ‘good or bad’ (p. 219). These markers often manifest through evaluative adjectives, adverbs, and ‘affective’ mental process verbs, which cluster into semantic sets that vary in intensity. In the excerpts above, evaluative adjectives such as ‘fundamental’ and ‘central’ reflect a high intensity of importance or desirability. In contrast, the evaluative noun ‘orientation’ suggests a lower level of intensity. These evaluation and modality markers imply that the role of HE in training socialism builders and inheritors is assigned a higher degree of desirability and prominence within the discourse.

## Discussion

A CDA of the Double First-Class Project policy texts reveals that the discourse of high-level talents is a hybrid discourse where the discourse of human capital and that of socialism builders and inheritors are mixed together. This analysis uncovers a ‘global/local dialectic’ in the recontextualisation of human capital discourse within Chinese HE policy texts. First, there is a ‘colonisation’ of local political language by the ‘global’ human capital discourse (Fairclough, 2003, p. 103). Chinese HE policy discourses reflect a utilitarian approach to evaluating education, echoing criticisms from liberal educationalists of the narrow economic focus of HCT (Gillies, 2017). Disciplines with high cultural value but limited vocational application face significant pressure due to their ambiguous link to economic outputs, leading to the devaluation of their teaching and research (Marginson, 1989, p. 24). In China, liberal arts disciplines are notably absent from the human capital discourse. Meanwhile, philosophy and social science disciplines align more closely with the discourse of socialism builders and inheritors.

Second, this ‘colonisation’ process involves an appropriation of the global discourse by integrating it with pre-existing local themes (Fairclough, 2003). To promote innovation-driven economic development in China, high-level talents are reworded to emphasise their scientific and technological innovation capacity. This suggests a local adaptation of the global human capital discourse, specifically the discourse of science and technology human talents, where investment in particular science and technology HE fields is anticipated to generate returns for national economic development and individual labour market outcomes. The process of appropriation demonstrates the CCP’s strategic connection of the discourse of human capital with the national macro-strategy of building China into a scientific superpower (Xi, 2017, p. 292). This finding supports Liu’s (2016) argument that the CCP has mobilised elite human resources to serve national development (p. 94). It also corroborates Han and Xu’s (2019) observation that the CCP employs policy tools to channel talent into specific disciplines—natural and applied sciences, particularly high-tech disciplines (p. 939).

In the traditional discourse of socialist builders and inheritors, morality is constantly emphasised, with ‘to build a strong character’ as a recurrent theme. The two master discourses entail different policy priorities: producing human capital to drive economic development on one hand, and fostering socialism builders and inheritors to advance socialism with Chinese characteristics on the other. The discourse of socialism builders and inheritors defines high-level talents in ways foreign to the human capital discourse. In the human capital discourse, ‘homo economicus’ is seen as a rational agent, ‘a non-moral person, if not immoral’, constrained only by factors such as time, money, and information (Tan, 2014, p. 432). In contrast, socialism builders and inheritors are heavily governed by moral values, particularly patriotism.

While the two discourses appear fundamentally incompatible in how they represent high-level talents, this study reveals that the CCP engages in ‘relational work’ to craft a hybrid discourse under the banner of ‘high-level talents’ (Fairclough, 2003, p. 101). A closer interdiscursive analysis shows that the discourse of socialism builders and inheritors is given primary importance, with the human capital discourse legitimated in terms of the discourse of socialism builders and inheritors. This synthesis is achieved through the bridging term—the Chinese Dream—which encourages young people to align their personal dreams with the broader Chinese Dream, framing their self-interest through the lens of nationalism (Vickers & Zeng, 2017).

## Conclusion

One notable contribution of this study is its demonstration of how the CCP government leverages the discourse of high-level talents in its HE policy texts to shape and sustain particular assumptions and practices within an increasingly globalised educational policy field. Contrary to some studies (Feher, 2009; Sellar & Zipin, 2019) that highlight the neoliberal trend of expanding the meaning of human capital, no evidence of an image of ‘entrepreneurs’ with saleable skills and attributes can be detected in the discourse of high-level talents. Instead, the findings align with Saich’s (2004) observation of ‘the paternalistic nature of the authoritarian party’ and its ‘policy of ‘infantilisation’ of society’:

Individuals were treated as children who did not know what was in their own best interests. Senior and local officials felt it their role not only to represent the population, but also to think on their behalf and take all important decisions in their interests (p. 219).

Within the hybrid discourse of high-level talents, ‘homo economicus’ is treated as children in need of the Party-state’s parental guidance through political socialisation. Moral values, such as patriotism and collectivism, are framed as ‘fundamental’ to individual success and are thus incorporated into the broader meaning of human capital. This study confirms that the CCP adopts a self-conscious strategy to reposition ‘high-level talents’ with ‘Chinese features’ to resist transnational pressures to conform to global policy agendas.

The findings contribute to a growing body of literature (e.g. Arnott & Ozga, 2010; Ozga & Jones, 2006; Wodak & Fairclough, 2010) that explores the capacity of ‘local’ contexts to negotiate or rearticulate emerging global educational policy discourses. Additionally, this study enhances our understanding of the application of CDA to Chinese text analysis. It highlights the importance of examining a nation’s political and ideological contexts within the field of educational research.

Furthermore, analysing the causal effects of the high-level talent discourse, particularly regarding science and technology human capital, deepens our understanding of the Party-State’s interventionist policies aimed at directing talents into certain disciplinary fields. Drawing on Fairclough’s (2003) accounts of the ‘dialectic of discourse’, the particular social imaginary can be enacted in students’ and their families’ ways of action/interaction (e.g. HE choice practice) and inculcated in their identities (ways of being). This discourse can thus be seen as ideological, contributing to the maintenance of ‘power relations and inequalities in producing social wrongs’ (Fairclough, 2003, p. 8). The ‘social wrong’ in this context refers to the suppression of students’ HE choices in favour of the national strategy and the Party-State’s governing power. Less utility-oriented disciplines, such as philosophy, may be considered ‘an expensive indulgence’, particularly for students from socio-economically disadvantaged backgrounds (Marginson, 1989: 4; Reay, 2017). However, further empirical studies are needed to examine the causal (or ideological) effects of these discourses.

## Data Availability

The author confirms that all data generated or analysed during this study are included in this published article. Furthermore, research data (policy texts) supporting the findings of this study are all publicly available, as shown in Table 1 of this article.

## Footnotes

1 Founded in 1952, the press is currently one part of the China Foreign Languages Publication and Distribution Administration and is owned and controlled by the CCP. The press published books targeting the international community. The collection is an authoritative source on Xi Jinping Thought, edited by the State Council Information Office, the Party Literature Research Office (of the Central Committee), and the China International Publishing Group. The collection focuses on Xi’s political philosophy related to political issues concerning China including economics, domestic politics, international relations, infrastructure, technology, environmentalism, peaceful co-existence, and the military.

2 Nation refers to a whole Chinese nation consisting of 56 ethnic groups, it also refers to ‘the people’.
